# Plasma‐Based Genomic Features Influencing Outcomes of T790M‐Positive Non–Small Cell Lung Cancer Receiving Osimertinib

**DOI:** 10.1002/cam4.71319

**Published:** 2025-11-12

**Authors:** Heng Liu, Junrong Yan, Junjun He, Rixu Lin

**Affiliations:** ^1^ Zhejiang Provincial Key Laboratory of Pancreatic Disease, the First Affiliated Hospital, School of Medicine Zhejiang University Hangzhou China; ^2^ Medical Department Nanjing Geneseeq Technology Inc. Nanjing China; ^3^ Department of Pathology The First Affiliated Hospital of Wenzhou Medical University Wenzhou China

**Keywords:** clinical outcomes, ctDNA, *EGFR* T790M, NSCLC, osimertinib

## Abstract

**Background:**

Circulating tumor DNA (ctDNA) provides a noninvasive method to clarify patients' genomic alterations. This study evaluated plasma‐derived ctDNA before second‐line osimertinib administration to explore the relationships between genomic alterations and clinical outcomes in patients with advanced non‐small‐cell lung cancer (NSCLC).

**Methods:**

We included 64 advanced NSCLC patients with EGFR T790M receiving second‐line osimertinib. Targeted DNA sequencing was conducted on plasma samples, and both clinical and genomic characteristics were assessed for the correlation with clinical outcomes.

**Results:**

Female patients showed longer progression‐free survival (PFS) than male patients. Smokers exhibited shorter PFS and overall survival (OS) than nonsmokers. EGFR exon 19 deletion (E19Del) tended to have improved PFS compared with L858R. Plasma T790M abundance was not associated with the response to osimertinib, PFS, or OS. Patients with TP53 mutations experienced significantly worse PFS than wild‐type ones. A significant PFS reduction was observed in patients with a blood tumor mutational burden (bTMB) ≥ 8, compared to those with a bTMB ⟨ 8 mut./Mb. The EGFR driver mutation type (E19Del versus L858R) and TP53 mutation status were independent factors influencing PFS by multivariate survival analysis. Furthermore, the combination of EGFR driver type and TP53 mutation status improved the predictive performance for clinical outcomes, including objective response rate (ORR) and PFS, but not OS.

**Conclusion:**

This study identified potential molecular features via ctDNA sequencing that were correlated to clinical outcomes in EGFR T790M‐positive advanced NSCLC treated with second‐line osimertinib.

## Graphical abstract

This study identified potential molecular features via ctDNA sequencing that were correlated to clinical outcomes in EGFR T790M‐positive advanced NSCLC treated with second‐line osimertinib. The combination of EGFR driver type and TP53 mutation status improved the predictive performance for clinical outcomes, including objective response rate (ORR) and PFS.

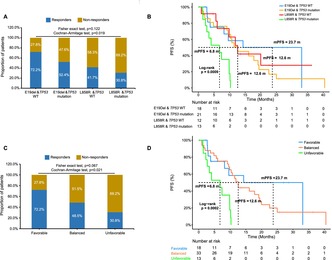

## 1 Introduction

Epidermal growth factor receptor (*EGFR*) mutation is recognized as one of the most important targets in patients with advanced non–small cell lung cancer (NSCLC), and EGFR tyrosine kinase inhibitor (EGFR‐TKI) administration is the standard treatment for NSCLC patients carrying *EGFR* sensitive mutations. Osimertinib, a third‐generation EGFR‐TKI, is now used as a first‐line strategy for *EGFR*‐positive NSCLC patients [1]. Based on the findings of the AURA series studies [2, 3, 4], osimertinib is also the standard care in NSCLC harboring *EGFR* T790M mutation after disease progression on the treatment of first‐ or second‐generation EGFR‐TKI.

Most patients will eventually develop resistance after undergoing first‐ or second‐generation EGFR‐TKI treatment, with the acquired *EGFR* T790M mutation being the most common resistance mechanism, accounting for approximately 50%–60% of cases [5, 6, 7]. However, conducting tumor rebiopsies to detect the T790M mutation in patients with advanced NSCLC who have been treated with EGFR‐TKIs is not always feasible in clinical practice. Circulating tumor DNA (ctDNA) provides a noninvasive approach for molecular diagnosis, helping to elucidate patients' genomic alterations and actionable information [8, 9, 10].

Although osimertinib is effective as a second‐line administration for patients with *EGFR* T790M‐positive NSCLC, a considerable number of patients experienced limited responses, and the clinical outcomes for some were not satisfactory [2, 4, 11]. Previous studies have indicated that various clinical and molecular factors can influence the clinical outcomes of such patients, such as age, smoking history, progression‐free survival (PFS) of the first‐generation EGFR‐TKI, and certain genomic characteristics [11, 12, 13, 14, 15]. Jin et al. performed targeted DNA sequencing on tumor tissue or pleural effusion samples and found that *PARP1* mutation, *MYC* amplification, and a higher tumor mutational burden (TMB) were negative predictive biomarkers for PFS in *EGFR* T790M‐positive NSCLC receiving second‐line osimertinib treatment [11]. However, the relationship between genomic alterations detected by ctDNA and clinical outcomes remains largely unresolved. Given that plasma is a noninvasive and readily accessible material with the highest feasibility for clinical use, especially in patients who have undergone front‐line treatment, it is crucial to investigate the correlation between the molecular characteristics of plasma ctDNA and the clinical outcomes, as well as to identify potential predictive markers of treatment efficacy.

In this study, we retrospectively collected 64 advanced NSCLC patients with the *EGFR* T790M mutation who received second‐line osimertinib after disease progression on first‐generation EGFR‐TKI treatment. Targeted DNA sequencing covering 425 cancer‐related genes was performed on the plasma‐derived ctDNA before osimertinib administration, and genomic features were elucidated. Clinical information and treatment outcomes, including radiographic response, PFS, and overall survival (OS) of osimertinib administration, were collected, and both clinical and genomic features were investigated for the correlation with clinical outcomes.

## 2 Method

### 2.1 Patient Enrollment

This study enrolled 64 histologically confirmed NSCLC patients who developed EGFR T790M‐mediated resistance following first‐generation EGFR‐TKI therapy. Key inclusion criteria comprised: (1) Histologically/cytologically verified NSCLC with measurable lesions (RECIST v1.1); (2) Advanced/metastatic disease (AJCC 8th edition staging); (3) Aged ≥ 18 years with disease progression following first‐line EGFR‐TKI therapy, including icotinib or gefitinib; (4) T790M positivity (plasma ctDNA; pleural effusion/other fluid positivity allowed if plasma‐negative); (5) Receipt of osimertinib as second‐line treatment; (6) Availability of clinicopathological data prior to osimertinib initiation; (7) Complete treatment response records (overall response rate [ORR], PFS, OS). Baseline clinical information, including age, sex, smoking history, and sites of metastasis, was collected. Treatment outcomes (ORR, PFS, and OS) were assessed during osimertinib therapy. This study was approved by the Ethics Committee of The First Affiliated Hospital of Wenzhou Medical University, and all enrolled patients provided informed consent.

### 2.2 Sample Processing and Next‐Generation Sequencing (NGS)

Plasma was extracted from 8 to 10‐mL peripheral blood in EDTA‐coated tubes within 4 h of blood collection. Circulating cell‐free DNA (cfDNA) from plasma was extracted using the Circulating Nucleic Acid Kit (QIAGEN). White blood cells (WBCs) were also collected from each patient and used as the normal control to distinguish germline variations, and WBC‐derived genomic DNA was extracted using the DNeasy Blood & Tissue Kit (Qiagen). Targeted NGS was conducted in Zhejiang Provincial Key Laboratory of Pancreatic Disease, The First Affiliated Hospital, College of Medicine, Zhejiang University (Hangzhou, China) or Department of Pathology, The First Affiliated Hospital of Wenzhou Medical University (Wenzhou, China). Library construction and sequencing were performed as previously described [16]. Briefly, the DNA underwent end repair, A‐tailing, and adaptor ligation, followed by polymerase chain reaction (PCR) and purification. Customized xGen lockdown probes (Integrated DNA Technologies) were employed for hybridization enrichment targeting 425 cancer‐relevant genes (Geneseeq). Captured libraries were quantified using the KAPA Library Quantification kit (KAPA Biosystems) and sequenced on Hiseq 4000 platforms (Illumina) to achieve targeted mean coverage depths of at least 5000X.

### 2.3 Bioinformatics Analysis

Bioinformatics analysis was also performed according to the previous study [17]. Briefly, sequencing data were demultiplexed by bcl2fastq (v2.19) and filtered by Trimmomatic. The data were then aligned to the hg19 reference human genome with the Burrows‐Wheeler Aligner (bwa‐mem) and further processed using the Picard suite and the Genome Analysis Toolkit (GATK). Single nucleotide polymorphisms (SNPs) and insertions or deletions were called by VarScan2 and HaplotypeCaller/UnifiedGenotyper in GATK, with a mutant allele frequency (MAF) cutoff of 0.1%. Common variants were removed using dbSNP and the 1000 Genome project. Germline mutations were filtered out by comparing against the patient's WBCs controls. Gene fusion was identified using FACTERA and copy number variation (CNV) was analyzed with ADTEx. The log2 ratio cut‐off for copy number gain was set at 1.6, while a log2 ratio cut‐off of ≤ 0.6 indicated copy number loss. Blood TMB (bTMB) was defined as the total number of all somatic base substitutions per megabase, including nonsynonymous and synonymous alterations, and indels in the coding region with the exception of known hotspot mutations in oncogenic driver genes and truncations in tumor suppressors, just as previously reported [18].

### 2.4 Statistical Analysis

Quantitative data are presented as median (range) or number of patients (percentage). Differences in *EGFR* T790M abundance between groups of response to osimertinib were measured by the Kruskal–Wallis test or Wilcoxon rank‐sum test. Comparisons of proportion between groups were analyzed using the Fisher's exact test, and the trend of ORR was assessed by the Cochran–Armitage test. The association of PFS or OS with the *EGFR* T790M abundance was determined by the Pearson correlation coefficient. Survival analysis was conducted with Kaplan–Meier curves, and the *p* value was determined with the log‐rank test, and the hazard ratio (HR) was calculated by the Cox proportional hazards model. Univariable or multivariate Cox regression was used to investigate the correlations between various variables and PFS or OS, with results presented as HRs and their 95% confidence interval (CI). A two‐sided *p* value of < 0.05 was considered significant for all tests. All analyses were performed using IBM SPSS Statistics version 25.

## 3 Results

### 3.1 Patient Characteristics and Treatment Efficacy

**FIGURE 1:**
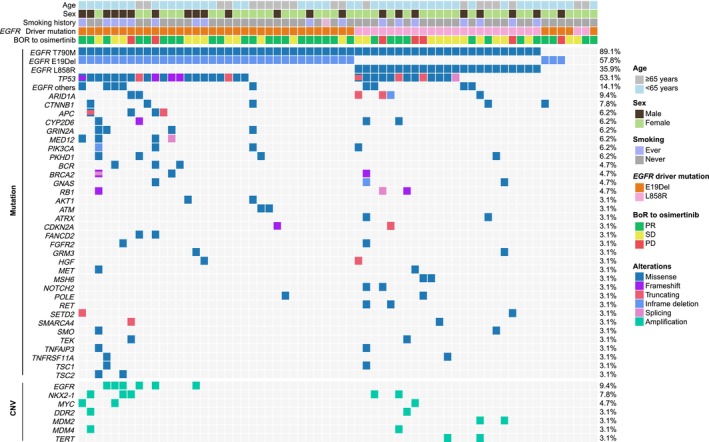
Genomic landscape of 64 advanced NSCLC patients who underwent plasma‐derived ctDNA sequencing. The oncoprint showing the pretreatment somatic mutations and CNVs that were detected in two or more cases. Abbreviations: BOR, best overall response; CNV, copy number variation; E19Del, exon 19 deletion; PD, progressive disease; PR, partial response; SD, stable disease.

A total of 64 advanced NSCLC patients with *EGFR* T790M‐positive receiving second‐line osimertinib were included in the study. All patients had received first‐generation EGFR‐TKI treatment and subsequently experienced disease progression (PD). Among them, 45 patients received icotinib and 19 patients received gefitinib as first‐line therapy. Of these, 57 patients exhibited T790M positivity in plasma sequencing (Figure 1), whereas the remaining seven cases, though plasma‐negative, were identified as T790M‐positive through pleural effusion liquid biopsy. Their pretreatment clinical characteristics are summarized in Table 1. All patients were diagnosed as adenocarcinoma (ADC). The median age was 61 years (range: 38–81), and 65.6% (42/64) were female, as well as 73.4% (47/64) having a history of smoking. Among them, 60.9% (39/64) originally harbored *EGFR* exon19 deletion (E19Del), while the remaining 39.1% carried L858R mutation. The most common metastatic organ was bone (53.1%). Other sites of metastasis included lymph node (18.8%), the central nervous system (CNS) (17.2%), liver (14.1%), and adrenal glands (3.1%).

**TABLE 1:** Clinical characteristics of NSCLC patients involved in this study before osimertinib treatment.

Characteristics	Number (%)
Total	64 (100%)
Median age, years (range)	61 (38–81)
Sex	
Male	22 (34.3%)
Female	42 (65.6%)
Smoking history	
Ever	17 (26.6%)
Never	47 (73.4%)
*EGFR* driver mutation	
E19Del	39 (60.9%)
L858R	25 (39.1%)
Histology	
ADC	64 (100%)
SCC	0 (0%)
CNS metastasis	
Yes	11 (17.2%)
No	53 (82.8%)
Liver metastasis	
Yes	9 (14.1%)
No	55 (85.9%)
Bone metastasis	
Yes	34 (53.1%)
No	30 (46.9%)
Lymph node metastasis	
Yes	12 (18.8%)
No	52 (81.2%)
Adrenal glands metastasis	
Yes	2 (3.1%)
No	62 (96.9%)
Pleural effusion	
With	37 (57.8%)
Without	27 (42.2%)
Best of response to osimertinib	
PR	33 (51.6%)
SD	24 (37.5%)
PD	7 (10.9%)

Abbreviations: ADC, adenocarcinoma; CNS, central nervous system; E19Del, exon 19 deletion; NSCLC, non–small cell lung cancer; PD, progressive disease; PR, partial response; SCC, squamous cell carcinoma; SD, stable disease.

Among the 64 patients who were detected with the T790M mutation by plasma or pleural effusion sequencing, the ORR was 51.6%, and the disease control rate (DCR) was 89.1%, including 33 (51.6%) patients who achieved a partial response (PR), 24 (37.5%) patients who showed stable disease (SD), and seven (10.9%) patients who experienced PD (Table 1). Patients who achieved PR were classified as responders, while those with SD or PD were categorized as nonresponders. The median PFS (mPFS) was 10.3 months, while the median OS (mOS) has not yet been reached (Figure S1).

### 3.2 Genomic Alterations Detected by Plasma‐Derived ctDNA Sequencing

All plasma samples from patients who were progressing while undergoing first‐generation EGFR‐TKI treatment and before osimertinib administration successfully underwent NGS testing. The mutational landscapes are shown in Figure 1, showcasing the somatic mutations or CNVs that were detected in two or more cases. Among them, only four did not exhibit any genomic alterations in their plasma. Additionally, three patients did not show T790M in the plasma, resulting in a total of seven patients who were T790M negative by ctDNA sequencing. *EGFR* E19Del and L858R were observed in 57.8% and 35.9% of the cases, respectively. The other site mutations of *EGFR* were found in nine (14.1%) patients, including L62R, P272H, G729A, E746V, P753S, L747S, G796D, R889G, M825I. In addition to *EGFR* mutations, the *TP53* gene exhibited the highest frequency, altering in 34 (53.1%) cases. Other relatively common concomitant somatic mutations included *ARID1A* (9.4%) and *CTNNB1* (7.8%). Regarding CNV, amplifications of *EGFR*, *NKX2‐1*, and *MYC* genes were noted in three or more cases. Besides, amplifications of *DDR2*, *MDM2*, *MDM4*, and *TERT* genes were also found in the study cohort.

### 3.3 Clinical Features Analysis of Treatment Efficacy to Osimertinib

**FIGURE 2:**
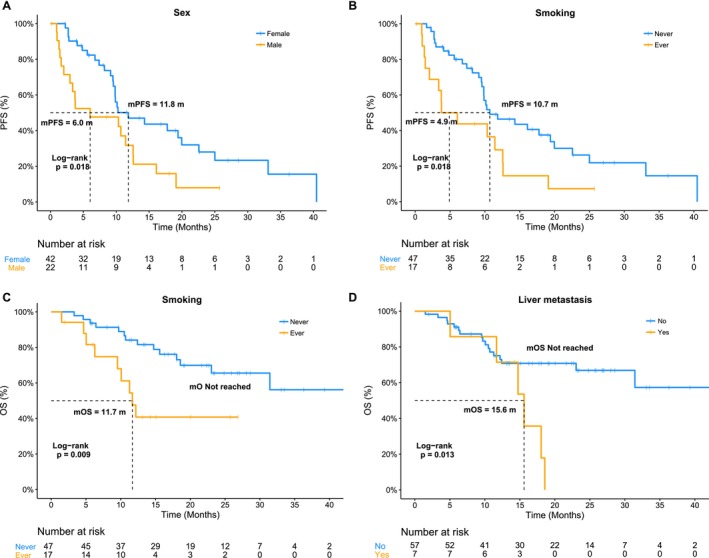
Clinical features analysis of treatment efficacy to osimertinib. (A) Kaplan–Meier estimates for progression‐free survival of females and males; (B‐C) Comparison of progression‐free survival and overall survival between smokers and nonsmokers; (D) Kaplan–Meier curve for overall survival of patients with liver metastasis and those without. Abbreviations: mOS, median OS; MPFS, median PFS.

The relationships between clinical features and treatment efficacy were analyzed, including age, sex, smoking history, tumor metastatic sites, and the occurrence of pleural effusion. No significant differences in these characteristics were observed between responders and nonresponders (data not shown). Female patients exhibited significantly longer mPFS (11.8 vs. 6.0 months, *p* = 0.018, Figure 2A & Table S1) and a trend toward better mOS (*p* = 0.093) compared to males (Table S1). Patients who had a smoking history showed worse mPFS (4.9 vs. 10.7 months, *p* = 0.018) and mOS (11.7 m vs. not reached, *p* = 0.009) than nonsmokers (Figure 2B,C & Table S1). Cases with liver metastasis experienced shorter mOS than those without liver metastasis (15.6 months vs. not reached, *p* = 0.013, Figure 2D & Table S1). Furthermore, the other metastatic sites were not associated with mPFS and mOS, including bone, CNS, and lymph node, and the occurrence of pleural effusion was not associated with either mPFS or mOS (Table S1).

Furthermore, we analyzed the association between clinical characteristics and both PFS and OS in the 57 patients with plasma‐detected T790M positivity, with results presented in Table S2. Similar to the findings in all 64 patients, female patients showed a trend toward longer mPFS (*p* = 0.059) and mOS (*p* = 0.091). Smokers had significantly shorter mPFS (*p* = 0.029) and mOS (*p* = 0.019) compared to nonsmokers. Patients with liver metastases had a shorter mOS than those without liver metastases (*p* = 0.025), although there was no difference in PFS.

### 3.4 Relationships Between ctDNA‐Derived T790M and Clinical Outcomes of Osimertinib

**FIGURE 3:**
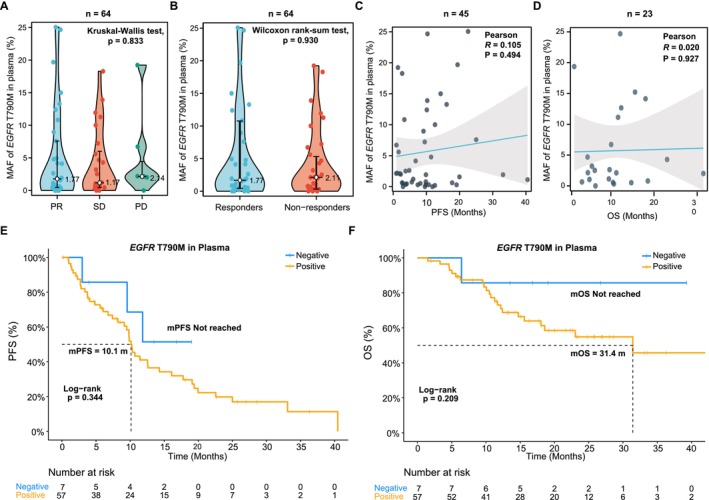
Relationships between ctDNA‐derived T790M and clinical outcomes of osimertinib. (A‐B) Comparison of plasma T790M abundance among different groups according to the response to osimertinib; (C‐D) The relationship between progression‐free survival or overall survival and plasma T790M abundance in patients who had already experienced progression‐free survival or overall survival events; (E‐F) Comparison of progression‐free survival and overall survival between T790M‐positive and T790M‐negative patients by ctDNA sequencing. Abbreviations: MAF, mutant allele frequency; mOS, median OS; mPFS, median PFS; PD, progressive disease; PR, partial response; SD, stable disease.

We investigated the relationship between the MAF of plasma T790M and clinical outcomes. In the PR, SD, or PD groups, the T790M MAF did not show a significant difference (*p* = 0.833), with median values of 1.77%, 1.17%, and 2.14%, respectively (Figure 3A). Furthermore, there was no significant difference in T790M MAF between responders and nonresponders (*p* = 0.930), with median values of 1.77% and 2.11%, respectively (Figure 3B). Additionally, we analyzed the relationship between PFS or OS and the abundance of T790M in patients who had already experienced PFS (*n* = 45) or OS (*n* = 23) events. The Pearson correlation test showed no significant correlations between plasma MAF of T790M and PFS (*r* = 0.105, *p* = 0.494) or OS (r = 0.020, *p* = 0.927) (Figure 3C,D). Besides, as mentioned above, no T790M mutation was detected in the plasma of seven patients; we then assessed the differences in PFS and OS between these seven patients and the remaining 57 patients. No difference was observed in both PFS (*p* = 0.344) and OS (*p* = 0.209, Figure 3E,F).

### 3.5 Correlations Between Pretreatment Genomic Features and Clinical Outcomes

**FIGURE 4:**
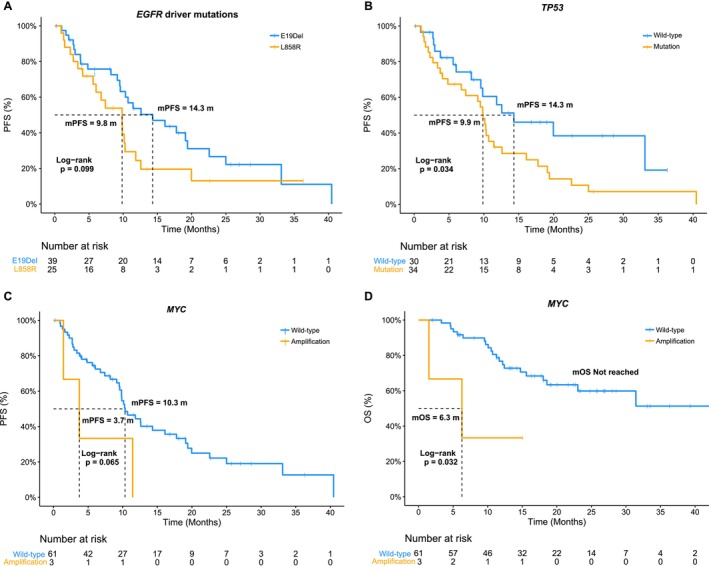
Correlations between pretreatment genomic features and clinical outcomes. (A) Kaplan–Meier curve for progression‐free survival of patients with *EGFR* E19Del and those with L858R; (B) Comparison of progression‐free survival and overall survival between cases that harbored *TP53* mutations and those that did not; (C) Overall survival stratified by the amplification status of the *MYC* gene. (D) Comparison of progression‐free survival between patients with a bTMB ≥ 8 and those with a bTMB < 8 mut./Mb. Abbreviations: bTMB, blood tumor mutational burden; E19Del, exon 19 deletion; mOS, median OS; mPFS, median PFS; mut./Mb, mutations per megabase.

The correlations between response, PFS or OS, and the somatic mutations or CNV observed in at least three cases were analyzed. No genomic alterations were found to be associated with the response rate, although *EGFR* E19del showed a trend toward a higher response rate compared to L858R, and patients with *CTNNB1* mutations tended to have an increased response rate (Figure S2A,B). Regarding survival, patients with original *EGFR* E19Del had better mPFS (14.3 vs. 9.8 months, *p* = 0.099, Figure 4A & Table S1) but not mOS (*p* = 0.570, Table S1) compared to those with L858R. Cases with *TP53* mutations demonstrated significantly worse mPFS (9.9 vs. 14.3 months, *p* = 0.034, Figure 4B & Table S1) but not OS (Table S1). Cases harboring *PIK3CA* or *RB1* mutations exhibited a tendency toward decreased PFS, while those who had *CTNNB1* mutations exhibited a trend toward better OS (Table S1). Regarding CNV, patients with *MYC* amplification had significantly worse mOS (6.3 months vs. not reached, *p* = 0.032, Figure 4C & Table S1) and a decreased mPFS (Table S1). Due to the limited *MYC* amplification events (*n* = 3), the observed correlations with survival outcomes must be regarded as exploratory, warranting cautious interpretation and further validation.

Furthermore, bTMB was evaluated for each patient, and the median bTMB was 3.67 mut./Mb (Range: 0–62.5). We then assessed the associations between bTMB and PFS or OS by establishing consecutive bTMB cut‐off points (Table S3). When bTMB ≥ 8 mut./Mb, we observed a significant reduction in PFS, with a mPFS of 6.8 months compared to 11.8 months for cases with bTMB < 8 mut./Mb (*p* = 0.027, Figure 4D). Although the difference in OS was not statistically significant, patients with a bTMB < 8 mut./Mb showed a trend toward improved mOS (Table S3). However, we did not observe a significant difference in response rate between patients with bTMB ≥ 8 mut./Mb and those with bTMB < 8 mut./Mb (*p* = 0.232, data not shown).

Similarly, among the 57 patients with plasma T790M positivity, those harboring *EGFR* E19Del mutations had a longer mPFS compared to those with L858R mutations (*p* = 0.073), although there was no difference in OS (*p* = 0.507, Table S2). *TP53*‐mutant patients showed a trend toward shorter mPFS (*p* = 0.065), with no difference in OS. Notably, patients with *MYC* amplification had a significantly inferior mOS (*p* = 0.049) and a trend toward shorter mPFS (*p* = 0.090). For the eight patients without any non‐*EGFR* co‐mutations detected in plasma, both PFS (*p* = 0.583) and OS (*p* = 0.704) were nearly identical to those observed in patients with additional genomic co‐alterations (Table S2). Continuous bTMB threshold analysis also revealed that those with bTMB ≥ 8 mut./Mb had significantly inferior mPFS compared to patients with bTMB < 8 mut./Mb (*p* = 0.046, Table S4).

### 3.6 Multivariate Cox Regression Analysis of Genomic and Clinical Features

In the multivariate analysis, we selected the genomic and clinical factors with a *p* value < 0.100 from the univariate analysis (Table 2). These factors included sex, smoking history, the type of *EGFR* drivers, mutations in the *TP53*, *PIK3CA*, and *RB1* genes, *MYC* amplification, and bTMB for PFS. For OS, we considered sex, smoking history, bone metastasis, liver metastasis, *CTNNB1* mutation, and *MYC* amplification. The results of the multivariate analysis indicated that the type of *EGFR* drivers (L858R vs. E19Del, HR = 2.90, *p* = 0.005) and *TP53* mutation status (mutation vs. wild type, HR = 2.05, *p* = 0.050) were identified as independent factors related to PFS. However, no factors were observed to be independently associated with OS (Table 2). In the multivariate analysis of the 57 patients with plasma T790M positivity, similar to the results observed in the overall population, *EGFR* driver mutation type (L858R vs. E19Del, HR = 2.86, *p* = 0.009) and *TP53* mutation status (mutant vs. wild type, HR = 2.32, *p* = 0.034) were identified as independent factors associated with PFS. Only age (≥ 65 vs. < 65 years, HR = 2.96, *p* = 0.032) was identified as an independent factor associated with OS (Table S5).

**TABLE 2:** Univariate and multivariable Cox regression analysis of progression‐free survival and overall survival by pretreatment clinical and genomic characteristics in all patients (*n* = 64).

Characteristics	Progression‐free survival				Overall survival			
	Univariate analysis		Multivariable analysis		Univariate analysis		Multivariable analysis	
	HR (95% CI)	*p*	HR (95% CI)	*p*	HR (95% CI)	p	HR (95% CI)	*p*
Age (Years)								
≥ 65 vs. < 65	—	—	—	—	—	—	—	—
Sex								
Male vs. Female	2.07 (1.12–3.83)	0.018	2.33 (0.63–8.60)	0.205	2.03 (0.87–4.71)	0.093	0.83 (0.11–6.5)	0.857
Smoking history								
Ever vs. Never	2.13 (1.12–4.06)	0.018	1.01 (0.26–3.85)	0.989	3.00 (1.27–7.11)	0.009	2.63 (0.31–22.12)	0.374
*EGFR* driver								
L858R vs. E19Del	1.66 (0.90–3.04)	0.099	2.90 (1.38–6.07)	**0.005**	—	—	—	—
Bone metastasis								
Yes vs. No	—	—	—	—	2.13 (0.89–3.97)	0.081	1.65 (0.64–4.24)	0.300
Liver metastasis								
Yes vs. No	—	—	—	—	3.13 (1.21–8.12)	0.013	2.36 (0.83–6.66)	0.105
*TP53*								
Mutation vs. WT	1.94 (1.04–3.63)	0.034	2.05 (1–4.22)	**0.050**	—	—	—	—
*PIK3CA*								
Mutation vs. WT	2.77 (0.82–9.34)	0.087	3.23 (0.8–13.02)	0.099	—	—	—	—
*RB1*								
Mutation vs. WT	2.73 (0.82–9.05)	0.088	1.46 (0.37–5.7)	0.589	—	—	—	—
*CTNNB1*								
Mutation vs. WT	—	—	—	—	1.23e‐08 (0‐Inf)	0.095	0 (0‐Inf)	0.998
*MYC* CNV								
Amplification vs. WT	2.9 (0.88–9.55)	0.065	1.64 (0.34–7.85)	0.535	4.37 (1.00–19.1)	0.032	2.25 (0.42–12.16)	0.346
bTMB (mut./Mb)								
≥ 8 vs. < 8	2.18 (1.08–4.44)	0.027	1.27 (0.5–3.22)	0.622	—	—	—	—

*Note:* Bold typeface serves only to highlight statistical significance (*p* ≤ 0.05).

Abbreviations: bTMB, blood tumor mutational burden; CI, confidence interval; CNV, copy number variation; E19Del, exon 19 deletion; HR, hazard ratio; Inf, infinity; mut./Mb, mutations per megabase; vs, versus; WT, wild type.

### 3.7 Combination of *EGFR* Driver Type and TP53 Mutation Status Improved Outcome Prediction

**FIGURE 5:**
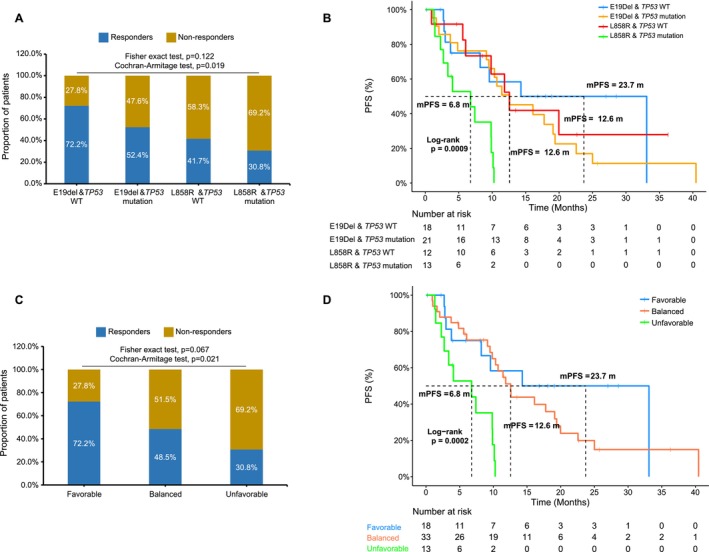
Combination of *EGFR* driver type and *TP53* mutation status improved predictive performance of clinical outcomes under osimertinib treatment. (A‐B) Comparison of response rate and progression‐free survival among four subgroups according to the variant status of *EGFR* driver type and *TP53* mutation. (C‐D) Comparison of response rate and progression‐free survival among three subgroups when patients were divided into Favorable, Balanced, or Unfavorable subgroups. Abbreviations: E19Del, exon 19 deletion; mPFS, median PFS; WT, wild type.

Given that *EGFR* driver type and *TP53* mutation status are independent factors affecting PFS according to multivariate analysis, we aimed to combine these two factors to assess their predictive value for clinical outcomes. Firstly, all the patients were divided into four subgroups: E19Del & *TP53* wild type, E19Del & *TP53* mutation, L858R & *TP53* wild type, and L858R & *TP53* mutation. We observed a trend toward differences in the ORR among these four subgroups (Fisher's exact test, *p* = 0.122, Figure 5A), with a significant declining trend evaluated by the Cochran–Armitage test (*p* = 0.019, Figure 5A). The mPFS for these subgroups was 23.7, 12.6, 12.6, and 6.8 months, respectively (*p* = 0.0009, Figure 5B). Next, since both subgroups of E19Del & *TP53* mutation and L858R & *TP53* wild type had only one negative factor affecting PFS (either a *TP53* mutation or L858R) and exhibited similar PFS, we merged them and designated them as the Balanced subgroup. Additionally, the subgroup with E19Del & *TP53* wild type was referred to as the Favorable subgroup, while those with L858R & *TP53* mutation were labeled as the Unfavorable subgroup. As illustrated in Figure 5C, there was a trend suggesting differences in the ORR (*p* = 0.067), along with a significant decreasing trend identified by the Cochran–Armitage test (*p* = 0.021) among these three subgroups. Simultaneously, a notable difference in PFS was observed among these three subgroups, which were 23.7, 12.6, and 6.8 months, respectively (*p* = 0.0002, Figure 5D). These results demonstrated that combining the type of *EGFR* driver and the status of *TP53* mutation could improve the predictive performance for clinical outcomes, including ORR and PFS. However, no difference in OS was noted among these subgroups (Figure S3). However, in light of the limited sample size, these findings remain exploratory and require confirmation in larger, independent cohorts.

## 4 Discussion

*EGFR* T790M mutation is the most prevalent mechanism of acquired resistance in lung adenocarcinoma patients with *EGFR* sensitive mutation who have been treated with first‐ or second‐generation EGFR‐TKIs as their initial therapy, accounting for nearly 50%–60% [5, 6, 7]. For patients with acquired T790M mutations, osimertinib is currently the standard second‐line treatment option. Although osimertinib has demonstrated remarkable efficacy, a significant number of patients still experienced intrinsic resistance or unsatisfactory treatment outcomes [2, 4, 11]. In real‐world settings, the ORR of osimertinib as a second‐line treatment for patients with acquired T790M was only 53.5% [11]. Another meta‐analysis that included 3086 patients with advanced NSCLC across 11 studies reported an ORR of 58% [19]. The mPFS of these two studies ranged from 10.33 to 10.58 months, results that were similar to those of our study, which showed an ORR of 51.6% and a mPFS of 10.3 months. Therefore, exploring potential biomarkers related to the clinical outcomes of osimertinib in the second‐line setting for T790M‐positive advanced NSCLC remains critically important and could help us make better clinical strategies.

On the other hand, tissue rebiopsy after resistance to first‐line treatment presents challenges in clinical practice and is often not feasible. Plasma‐derived ctDNA provides a noninvasive and real‐time alternative. Numerous previous studies have demonstrated that ctDNA could be used to elucidate patients' genomic variations and provide actionable information to guide clinical treatment decisions [8, 11, 20, 21]. Herein, we explored potential biomarkers related to the outcomes of second‐line osimertinib treatment in patients with *EGFR* T790M‐mutated advanced NSCLC based on the genomic features obtained by ctDNA sequencing in conjunction with pretreatment clinical characteristics.

In this study, we discovered that PFS and OS in nonsmokers were significantly better than those in smokers, aligning with previous research [11, 12]. Furthermore, we observed that PFS was significantly higher in female patients compared to males, and there was also a trend suggesting better OS for females. In the population of this study, all the females were nonsmokers, while most of the males were smokers. This may have contributed to the association of both nonsmokers and females with improved outcomes in the univariate analysis. Although we did not find a significant correlation between age and PFS or OS, as indicated in previous studies [11, 13], our results suggested that younger patients exhibited a trend of having worse PFS and OS compared to older patients. This finding aligns with earlier reports and may be attributed to a limited sample size.

Additionally, we found that the original *EGFR* mutation type was correlated to the PFS, with E19Del exhibiting better PFS than L858R. Although the *p* value is not significant, likely due to the insufficient sample size, we can clearly observe the impact of the *EGFR* driver type on PFS, as previously reported [22, 23, 24]. Impressively, it emerged as an independent factor that significantly associated with PFS after conducting a multivariate analysis with other clinical or genomic features. Consequently, combining osimertinib with chemotherapy or antiangiogenic therapiesp may improve the treatment effectiveness for patients with the L858R mutation.

We also investigated the relationship between T790M abundance detected through ctDNA and the outcomes of osimertinib treatment. Consistent with previous studies [11], the abundance of the T790M mutation did not show a significant correlation with responses, PFS, or OS. In our study, T790M was detected in the plasma of the vast majority of patients (89%); the ORR was 51.3%, and the mPFS was 10.3 months, while the mOS had not yet been reached, which aligns with the efficacy observed in an earlier study using tissue or pleural effusion samples [11]. Additionally, there was no discernible difference in PFS and OS between patients with T790M and those without T790M in plasma. These findings suggest that detecting T790M via ctDNA is a viable alternative for guiding osimertinib treatment.

The clinical implications of plasma‐based detection of T790M are highly significant. Compared to tissue rebiopsy, plasma ctDNA testing is minimally invasive, repeatable, and suitable for patients with inaccessible lesions or poor performance status [25]. This feasibility advantage enables quicker identification of patients eligible for targeted therapies and reduces delays in initiating appropriate second‐line treatment. Furthermore, numerous studies have demonstrated high concordance between ctDNA and tissue‐based *EGFR* mutation testing, including for T790M [11, 21, 26, 27]; however, discordance may occur due to tumor heterogeneity, variations in ctDNA shedding, or technical limitations. Importantly, plasma‐based testing allows dynamic, real‐time monitoring of tumor genotypes and may detect resistance mutations before radiographic progression or when tissue is unobtainable. Nevertheless, limitations persist: the sensitivity can decrease with low tumor burden or reduced ctDNA shedding, leading to potential false negatives, particularly among patients with low tumor volume or indolent disease. Therefore, as recommended in prior studies [11, 28], repeat plasma testing or complementary analysis using tissue or pleural fluid samples should be considered in cases where clinical suspicion remains high despite negative plasma results. Despite these limitations, ctDNA‐based detection is increasingly incorporated into clinical guidelines as a practical tool for guiding EGFR‐TKI sequencing in NSCLC, and our findings further support its value for identifying actionable resistance mutations and stratifying patients for osimertinib therapy.

Notably, eight patients (8/57%, 14.0%) exhibited only *EGFR* sensitizing/T790M mutations without additional alterations, although their PFS was not different from that of patients without additional genomic alterations. Several potential explanations may account for this observation: (1) *EGFR*‐driven oncogenic dominance: These tumors may be predominantly driven by *EGFR* pathway alterations, with minimal contribution from other genomic events at sampling. Such cases could represent classic “oncogene‐addicted” tumors dependent on *EGFR* signaling, characterized by simpler genomic profiles with fewer bypass resistance mechanisms. (2) ctDNA shedding heterogeneity: Low tumor burden or reduced ctDNA release in some patients may limit subclonal alteration detection. (3) Technical detection limitations: assay sensitivity may be restricted by low ctDNA levels, panel breadth constraints, or sequencing depth, potentially missing off‐target/low‐frequency variants. (4) Evolutionary stage or nongenomic mechanisms: These cases may represent early resistance evolution with T790M‐dominant mechanisms or nongenomic adaptations (e.g., epigenetic/microenvironmental changes) undetectable by DNA sequencing. In summary, multiple factors could underlie this observation. Further studies using higher sensitivity platforms or longitudinal monitoring should clarify whether this reflects technical constraints or a distinct biologic subgroup.

Although T790M mutation is the predominant mechanism of acquired resistance to first‐ and second‐generation EGFR‐TKIs, previous studies indicated that other genomic variations often appear alongside T790M [11, 29, 30]. These co‐occurred genetic alterations may cooperate with T790M to affect the results of osimertinib administration [11, 14, 15]. In this study, we found that *TP53* mutation was associated with worse PFS, which was detected more than 50% of the cases. Furthermore, it was still an independent factor associated with PFS via multivariate analysis. *TP53* is the most critical tumor suppressor gene in human beings. Previous research have indicated that *TP53* mutations could impact the efficacy of EGFR‐TKI in patients with *EGFR*‐positive NSCLC, and the combination of antiangiogenesis or chemotherapy drugs with EGFR‐TKI has been shown to enhance treatment outcomes [14, 31, 32, 33, 34]. Furthermore, considering that *EGFR* driver type and *TP53* mutation status are independent factors influencing PFS based on the results of multivariate analysis, we combined these two factors to explore their predictive performance for clinical outcomes. The results indicated that the ORR and PFS were best for the E19Del & *TP53* wild‐type subgroup, whereas the L858R & *TP53* mutation subgroup had the worst ORR and PFS. The other two subgroups of E19Del & *TP53* mutation and L858R & *TP53* wild type fell in the middle, exhibiting comparable ORR and PFS. However, there were no differences in OS among these subgroups. These findings proved that the combination of *EGFR* driver type and *TP53* mutation status may enhance the predictive performance for ORR and PFS. This indicates that patients with both L858R and *TP53* mutations should be advised to combine chemotherapy or antiangiogenic drugs with osimertinib, or even consider using all of these treatments together in T790M‐mutated NSCLC. In contrast, patients with the E19Del and wild type *TP53* may be relatively safely treated with osimertinib alone. For those with either L858R or *TP53* mutation alone, it may be reasonable to consider combining osimertinib with other therapies, and clinicians should make optimal treatment decisions based on the individual patient's circumstances.

Additionally, our study indicated that a bTMB of 8 or higher was linked to poorer PFS. TMB is recognized as a significant positive predictive biomarker for the effectiveness of immune checkpoint inhibitors [35]. Previous research has shown that TMB emerged as a negative correlation with clinical outcomes in patients with advanced NSCLC harboring *EGFR* mutations who were treated with first‐ or second‐generation EGFR‐TKI [29, 36, 37, 38]. Notably, Jin et al. found that TMB evaluated through tissue or pleural effusion samples was inversely related to the PFS of patients receiving the third‐generation EGFR‐TKI, osimertinib [11]. However, it remains uncertain whether the bTMB derived from ctDNA sequencing can effectively predict the outcomes of second‐line treatment with osimertinib in T790M‐positive NSCLC patients. Our study made a preliminary exploration and found that TMB derived from ctDNA sequencing could deliver results comparable to those obtained from tissue detection. However, we employed a continuous gradient analysis to determine the optimal threshold for TMB, revealing that patients with TMB ≥ 8 had significantly poorer PFS compared to those with TMB < 8. This finding may be partially coincidental, and the interpretation of this optimal cutoff should be approached with caution, especially as more evidence is needed before it can be applied in clinical practice. In summary, further studies are necessary in the future to confirm the value of bTMB in clinical applications involving second‐line osimertinib. Of course, considering that the number of patients who receive osimertinib as a second‐line treatment may decrease, it is important to investigate the value of bTMB in the first‐line setting of osimertinib in *EGFR*‐positive NSCLC patients.

The study has several limitations that should be acknowledged. Firstly, the sample size was not large enough that may impact the generalizability and reliability of the findings. Secondly, as a single‐center study, the potential heterogeneity of the population may limit the applicability of our findings to other settings. While our study offers important insights into potential biomarkers of response and clinical outcomes, these findings are exploratory and require validation in larger, independent cohorts.

In conclusion, this study identified the T790M mutation using plasma‐derived ctDNA sequencing, which guided the use of osimertinib as a second‐line treatment, and determined potential genomic features associated with treatment outcomes. This demonstrates that ctDNA‐based liquid biopsies can guide subsequent clinical management and discover potential biomarkers that may influence clinical outcomes before treatment. Moreover, the combination of certain markers in this study enhanced the predictive performance of patient benefits. These exploratory findings may inform future strategies for patient stratification and treatment optimization, although validation in larger, independent cohorts is warranted to confirm their clinical relevance.

## Author Contributions

**Rixu Lin:** conceptualization (lead), supervision (equal), writing – review and editing (lead). **Heng Liu:** data curation (lead), formal analysis (lead), project administration (lead), writing – original draft (equal), writing – review and editing (supporting). **Junjun He:** conceptualization (equal), project administration (equal), supervision (lead), writing – review and editing (supporting). **Junrong Yan:** formal analysis (equal), visualization (lead), writing – review and editing (supporting).

## Ethics Statement

The procedures and protocol of this study were approved by the Medical Ethics Committee of The First Affiliated Hospital of Wenzhou Medical University (KY2025‐R004). Written informed consent for sample usage in research was obtained from each patient before sample collection.

## Conflicts of Interest

Junrong Yan is an employee of Nanjing Geneseeq Technology Inc. All other authors declared no conflicts of interest.

## Supporting information


**Figure S1.** The progression‐free survival and overall survival for all the patients enrolled in this study. Abbreviations: mPFS, median PFS; mOS, median OS.

**Figure S2.** Effects of the type of *EGFR* driver mutation or the status of *CTNNB1* mutation on the response rate to osimertinib treatment. Abbreviations: E19Del, exon 19 deletion.

**Figure S3.** Comparison of overall survival among different subgroups according to the status of *EGFR* driver type and *TP53* mutation. Abbreviations: E19Del, exon 19 deletion; WT, wild type.

**Table S1.** Univariate Cox analysis of progression‐free survival and overall survival based on pretreatment clinical characteristics and genomic alterations detected in at least three patients among all patients (*n* = 64).

**Table S2.** Univariate Cox analysis of progression‐free survival and overall survival in plasma T790M‐positive patients according to baseline clinical characteristics and genomic alterations (*n* = 57).

**Table S3.** Univariate Cox analysis of progression‐free survival and overall survival by establishing consecutive bTMB cut‐off points.

**Table S4.** Univariate Cox analysis of progression‐free survival and overall survival by establishing consecutive bTMB cut‐off points in plasma T790M‐positive patients (*n* = 57).

**Table S5.** Univariate and multivariable Cox regression analysis of progression‐free survival and overall survival by pretreatment clinical and genomic characteristics in plasma T790M‐positive patients (*n* = 57).

## Data Availability

The data that support the findings of this study are available on request from the corresponding author, [R.X. L]. The data are not publicly available due to containing information that could compromise the privacy of research participants.

## References

[1] J. C. Soria , Y. Ohe , J. Vansteenkiste , et al., “Osimertinib in Untreated EGFR‐Mutated Advanced Non‐Small‐Cell Lung Cancer,” New England Journal of Medicine 378 (2018): 113–125. 29151359 10.1056/NEJMoa1713137

[2] P. A. Janne , J. C. Yang , D. W. Kim , et al., “AZD9291 in EGFR Inhibitor‐Resistant Non‐Small‐Cell Lung Cancer,” New England Journal of Medicine 372 (2015): 1689–1699. 25923549 10.1056/NEJMoa1411817

[3] J. C. Yang , M. J. Ahn , D. W. Kim , et al., “Osimertinib in Pretreated T790M‐Positive Advanced Non‐Small‐Cell Lung Cancer: AURA Study Phase II Extension Component,” Journal of Clinical Oncology 35 (2017): 1288–1296. 28221867 10.1200/JCO.2016.70.3223

[4] T. S. Mok , Y. L. Wu , M. J. Ahn , et al., “Osimertinib or Platinum‐Pemetrexed in EGFR T790M‐Positive Lung Cancer,” New England Journal of Medicine 376 (2017): 629–640. 27959700 10.1056/NEJMoa1612674 PMC6762027

[5] H. A. Yu , M. E. Arcila , N. Rekhtman , et al., “Analysis of Tumor Specimens at the Time of Acquired Resistance to EGFR‐TKI Therapy in 155 Patients With EGFR‐Mutant Lung Cancers,” Clinical Cancer Research 19 (2013): 2240–2247. 23470965 10.1158/1078-0432.CCR-12-2246 PMC3630270

[6] C. S. Kuo , P. L. Su , Y. F. Wei , et al., “T790M Detection Rate After First‐Line Combination Therapy With Bevacizumab and EGFR‐TKIs in Advanced NSCLC (TERRA Study),” American Journal of Cancer Research 13 (2023): 3100–3112. 37559987 PMC10408489

[7] M. E. Arcila , G. R. Oxnard , K. Nafa , et al., “Rebiopsy of Lung Cancer Patients With Acquired Resistance to EGFR Inhibitors and Enhanced Detection of the T790M Mutation Using a Locked Nucleic Acid‐Based Assay,” Clinical Cancer Research 17 (2011): 1169–1180. 21248300 10.1158/1078-0432.CCR-10-2277 PMC3070951

[8] L. Keller , Y. Belloum , H. Wikman , and K. Pantel , “Clinical Relevance of Blood‐Based ctDNA Analysis: Mutation Detection and Beyond,” British Journal of Cancer 124 (2021): 345–358. 32968207 10.1038/s41416-020-01047-5 PMC7852556

[9] Y. Zhang , Y. Yao , Y. Xu , et al., “Pan‐Cancer Circulating Tumor DNA Detection in Over 10,000 Chinese Patients,” Nature Communications 12 (2021): 11. 10.1038/s41467-020-20162-8 PMC7782482 33397889

[10] C. Reina , B. Sabanovic , C. Lazzari , V. Gregorc , and C. Heeschen , “Unlocking the Future of Cancer Diagnosis—Promises and Challenges of ctDNA‐Based Liquid Biopsies in Non‐Small Cell Lung Cancer,” Translational Research 272 (2024): 41–53. 38838851 10.1016/j.trsl.2024.05.014

[11] Y. Jin , C. Lin , X. Shi , et al., “Impact of Clinical and Molecular Features on Efficacy and Outcome of Patients With Non‐Small Cell Lung Cancer Receiving Second‐Line Osimertinib,” BMC Cancer 22 (2022): 586. 35643428 10.1186/s12885-022-09683-1 PMC9145492

[12] J. Liu , X. Li , Y. Shao , X. Guo , and J. He , “The Efficacy and Safety of Osimertinib in Treating Nonsmall Cell Lung Cancer: A PRISMA‐Compliant Systematic Review and Meta‐Analysis,” Medicine (Baltimore) 99 (2020): e21826. 32846826 10.1097/MD.0000000000021826 PMC7447427

[13] Y. Kato , Y. Hosomi , K. Watanabe , et al., “Impact of Clinical Features on the Efficacy of Osimertinib Therapy in Patients With T790M‐Positive Non‐Small Cell Lung Cancer and Acquired Resistance to Epidermal Growth Factor Receptor Tyrosine Kinase Inhibitors,” Journal of Thoracic Disease 11 (2019): 2350–2360. 31372272 10.21037/jtd.2019.06.03 PMC6626778

[14] C. Aggarwal , C. W. Davis , R. Mick , J. C. Thompson , and S. Ahmed , “Influence of TP53 Mutation on Survival in Patients With Advanced EGFR‐Mutant Non‐Small‐Cell Lung Cancer,” JCO Precision Oncology 2 (2018): 1–29. 10.1200/PO.18.00107 PMC6372114 30766968

[15] Y. Zeng , Y. Feng , G. Fu , et al., “Acquired Concurrent EGFR T790M and Driver Gene Resistance From EGFR‐TKIs Hampered Osimertinib Efficacy in Advanced Lung Adenocarcinoma: Case Reports,” Frontiers in Pharmacology 13 (2022): 838247. 35462930 10.3389/fphar.2022.838247 PMC9020767

[16] B. Qiu , W. Guo , F. Zhang , et al., “Dynamic Recurrence Risk and Adjuvant Chemotherapy Benefit Prediction by ctDNA in Resected NSCLC,” Nature Communications 12 (2021): 6770. 10.1038/s41467-021-27022-z PMC8605017 34799585

[17] Y. Wang , W. Wang , T. Zhang , et al., “Dynamic bTMB Combined With Residual ctDNA Improves Survival Prediction in Locally Advanced NSCLC Patients With Chemoradiotherapy and Consolidation Immunotherapy,” Journal of the National Cancer Center 4 (2024): 177–187. 39282582 10.1016/j.jncc.2024.01.008 PMC11390629

[18] W. Fang , Y. Ma , J. C. Yin , et al., “Comprehensive Genomic Profiling Identifies Novel Genetic Predictors of Response to Anti‐PD‐(L)1 Therapies in Non‐Small Cell Lung Cancer,” Clinical Cancer Research 25 (2019): 5015–5026. 31085721 10.1158/1078-0432.CCR-19-0585

[19] L. Yi , J. Fan , R. Qian , P. Luo , and J. Zhang , “Efficacy and Safety of Osimertinib in Treating EGFR‐Mutated Advanced NSCLC: A Meta‐Analysis,” International Journal of Cancer 145 (2019): 284–294. 30613959 10.1002/ijc.32097 PMC6590181

[20] Y. Hong , W. Zhuang , J. Lai , et al., “Plasma EGFR Mutation ctDNA Dynamics in Patients With Advanced EGFR‐Mutated NSCLC Treated With Icotinib: Phase 2 Multicenter Trial Result,” Scientific Reports 14 (2024): 23115. 39367090 10.1038/s41598-024-73749-2 PMC11452669

[21] K. S. Thress , R. Brant , T. H. Carr , et al., “EGFR Mutation Detection in ctDNA From NSCLC Patient Plasma: A Cross‐Platform Comparison of Leading Technologies to Support the Clinical Development of AZD9291,” Lung Cancer 90 (2015): 509–515. 26494259 10.1016/j.lungcan.2015.10.004

[22] S. Gen , I. Tanaka , M. Morise , et al., “Clinical Efficacy of Osimertinib in EGFR‐Mutant Non‐Small Cell Lung Cancer With Distant Metastasis,” BMC Cancer 22 (2022): 654. 35698083 10.1186/s12885-022-09741-8 PMC9195197

[23] M. Sheng , F. Wang , Y. Zhao , et al., “Comparison of Clinical Outcomes of Patients With Non‐Small‐Cell Lung Cancer Harbouring Epidermal Growth Factor Receptor Exon 19 or Exon 21 Mutations After Tyrosine Kinase Inhibitors Treatment: A Meta‐Analysis,” European Journal of Clinical Pharmacology 72 (2016): 1–11. 26490356 10.1007/s00228-015-1966-0

[24] I. J. Z. Eide , A. Helland , S. Ekman , et al., “Osimertinib in T790M‐Positive and ‐Negative Patients With EGFR‐Mutated Advanced Non‐Small Cell Lung Cancer (The TREM‐Study),” Lung Cancer 143 (2020): 27–35. 32200138 10.1016/j.lungcan.2020.03.009

[25] C. Rolfo , P. Mack , G. V. Scagliotti , et al., “Liquid Biopsy for Advanced NSCLC: A Consensus Statement From the International Association for the Study of Lung Cancer,” Journal of Thoracic Oncology 16 (2021): 1647–1662. 34246791 10.1016/j.jtho.2021.06.017

[26] C. Karlovich , J. W. Goldman , J. M. Sun , et al., “Assessment of EGFR Mutation Status in Matched Plasma and Tumor Tissue of NSCLC Patients From a Phase I Study of Rociletinib (CO‐1686),” Clinical Cancer Research 22 (2016): 2386–2395. 26747242 10.1158/1078-0432.CCR-15-1260 PMC6886231

[27] C. Aggarwal , J. C. Thompson , T. A. Black , et al., “Clinical Implications of Plasma‐Based Genotyping With the Delivery of Personalized Therapy in Metastatic Non‐Small Cell Lung Cancer,” JAMA Oncology 5 (2019): 173–180. 30325992 10.1001/jamaoncol.2018.4305 PMC6396811

[28] T. Stockley , C. A. Souza , P. K. Cheema , et al., “Evidence‐Based Best Practices for EGFR T790M Testing in Lung Cancer in Canada,” Current Oncology 25 (2018): 163–169. 29719432 10.3747/co.25.4044 PMC5927787

[29] C. M. Blakely , T. B. K. Watkins , W. Wu , et al., “Evolution and Clinical Impact of Co‐Occurring Genetic Alterations in Advanced‐Stage EGFR‐Mutant Lung Cancers,” Nature Genetics 49 (2017): 1693–1704. 29106415 10.1038/ng.3990 PMC5709185

[30] Y. Jin , H. Bao , X. Le , et al., “Distinct Co‐Acquired Alterations and Genomic Evolution During TKI Treatment in Non‐Small‐Cell Lung Cancer Patients With or Without Acquired T790M Mutation,” Oncogene 39, no. 9 (2020): 1846–1859. 31754213 10.1038/s41388-019-1104-z

[31] Z. Zhang , J. Xue , Y. Yang , et al., “Influence of TP53 Mutation on Efficacy and Survival in Advanced EGFR‐Mutant Non‐Small Cell Lung Cancer Patients Treated With Third‐Generation EGFR Tyrosine Kinase Inhibitors,” MedComm (2020) 5 (2024): e586. 38832214 10.1002/mco2.586 PMC11144614

[32] B. Lan , N. Zhao , K. Du , and B. Leng , “Concurrent TP53 Mutations Predict a Poor Prognosis of EGFR‐Mutant NSCLCs Treated With TKIs: An Updated Systematic Review and Meta‐Analysis,” Oncology Letters 24 (2022): 384. 36238360 10.3892/ol.2022.13504 PMC9494618

[33] M. Nishio , L. Paz‐Ares , M. Reck , et al., “RELAY, Ramucirumab Plus Erlotinib (RAM+ERL) in Untreated Metastatic EGFR‐Mutant NSCLC (EGFR+ NSCLC): Association Between TP53 Status and Clinical Outcome,” Clinical Lung Cancer 24 (2023): 415–428. 37076395 10.1016/j.cllc.2023.02.010

[34] H. Sun , P. Ren , Y. Chen , et al., “Optimal Therapy for Concomitant EGFR and TP53 Mutated Non‐Small Cell Lung Cancer: A Real‐World Study,” BMC Cancer 23 (2023): 198. 36864384 10.1186/s12885-023-10637-4 PMC9979422

[35] S. J. Klempner , D. Fabrizio , S. Bane , et al., “Tumor Mutational Burden as a Predictive Biomarker for Response to Immune Checkpoint Inhibitors: A Review of Current Evidence,” Oncologist 25 (2020): e147–e159. 31578273 10.1634/theoncologist.2019-0244 PMC6964127

[36] M. Offin , H. Rizvi , M. Tenet , et al., “Tumor Mutation Burden and Efficacy of EGFR‐Tyrosine Kinase Inhibitors in Patients With EGFR‐Mutant Lung Cancers,” Clinical Cancer Research 25 (2019): 1063–1069. 30045933 10.1158/1078-0432.CCR-18-1102 PMC6347551

[37] C. Lin , X. Shi , J. Zhao , et al., “Tumor Mutation Burden Correlates With Efficacy of Chemotherapy/Targeted Therapy in Advanced Non‐Small Cell Lung Cancer,” Frontiers in Oncology 10 (2020): 480. 32411590 10.3389/fonc.2020.00480 PMC7201001

[38] J. Y. Sung , D. W. Park , and S. H. Lee , “High Tumor Mutation Burden Is Associated With Poor Clinical Outcome in EGFR‐Mutated Lung Adenocarcinomas Treated With Targeted Therapy,” Biomedicine 10, no. 9 (2022): 2109. 10.3390/biomedicines10092109 PMC9495802 36140210

