# Supplementary figures and images for: Plasma‐Based Genomic Features Influencing Outcomes of T790M‐Positive Non–Small Cell Lung Cancer Receiving Osimertinib
Source: Cancer Med. 2025 Nov 12;14(21):e71319. doi: 10.1002/cam4.71319 (PMC12605980; doi:10.1002/cam4.71319)

Figure S1

**A**

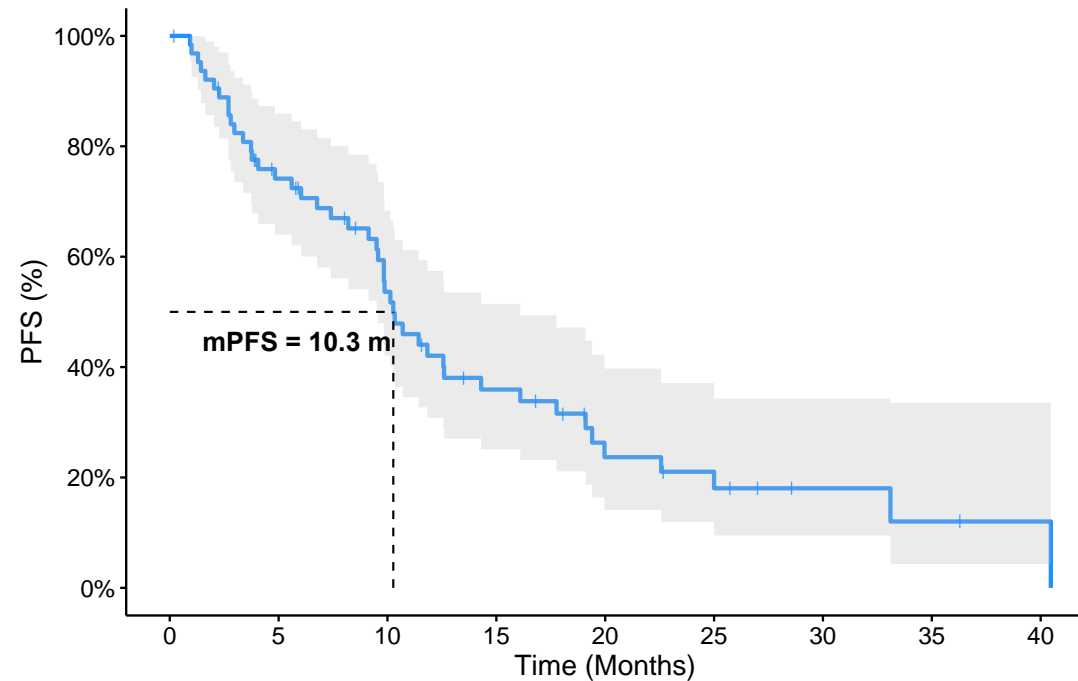

**B**

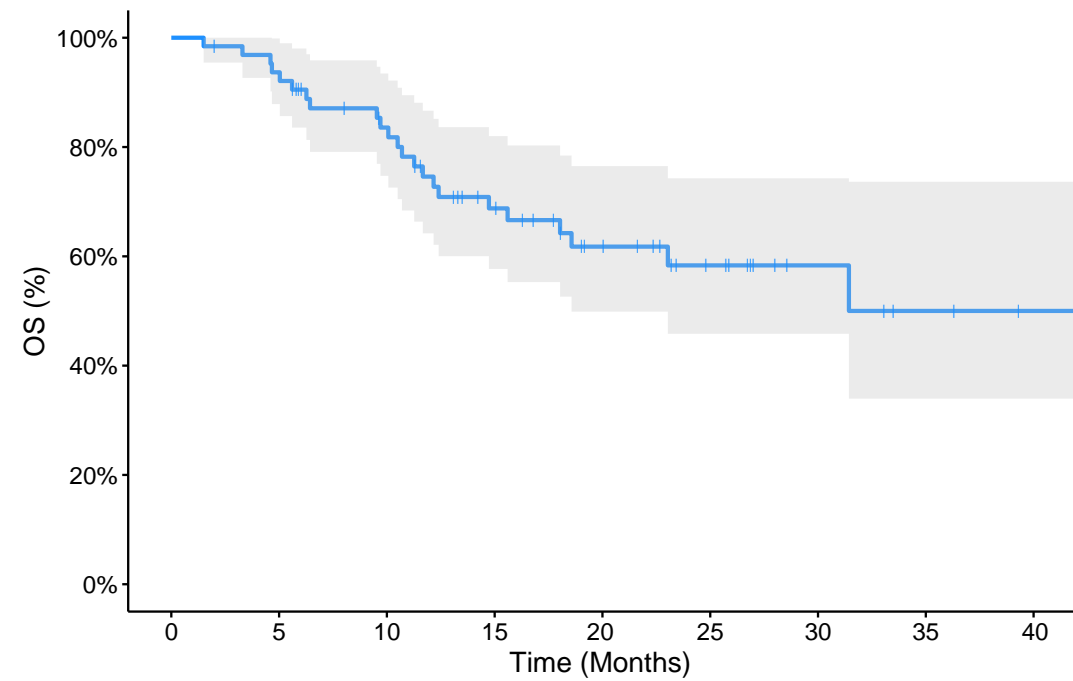

Supplement: Supplementary file 1 — Figure S1. The progression‐free survival and overall survival for all the patients enrolled in this study. Abbreviations: mPFS, median PFS; mOS, median OS. [file CAM4-14-e71319-s003.pdf]

Figure S2

**A**

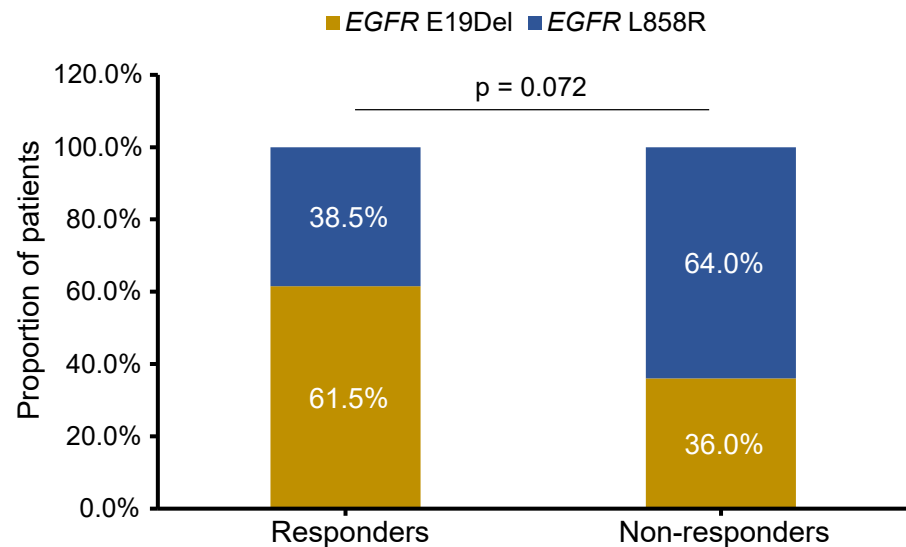

**B**

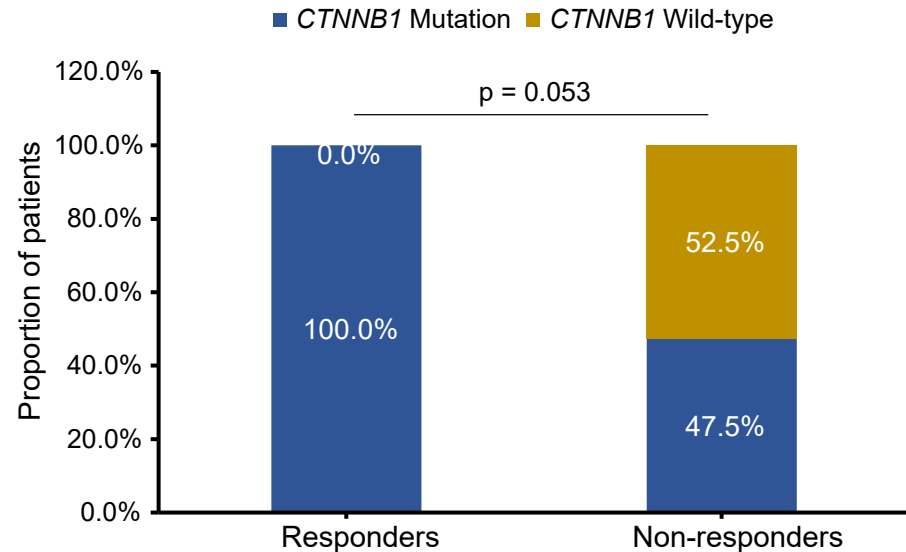

Supplement: Supplementary file 2 — Figure S2. Effects of the type of EGFR driver mutation or the status of CTNNB1 mutation on the response rate to osimertinib treatment. Abbreviations: E19Del, exon 19 deletion. [file CAM4-14-e71319-s006.pdf]
