# Supplementary material for: Plasma‐Based Genomic Features Influencing Outcomes of T790M‐Positive Non–Small Cell Lung Cancer Receiving Osimertinib
Source: Cancer Med. 2025 Nov 12;14(21):e71319. doi: 10.1002/cam4.71319 (PMC12605980; doi:10.1002/cam4.71319)
Supplement: Supplementary file 3 — Figure S3. Comparison of overall survival among different subgroups according to the status of EGFR driver type and TP53 mutation. Abbreviations: E19Del, exon 19 deletion; WT, wild type. [file CAM4-14-e71319-s002.pdf]

Figure S3

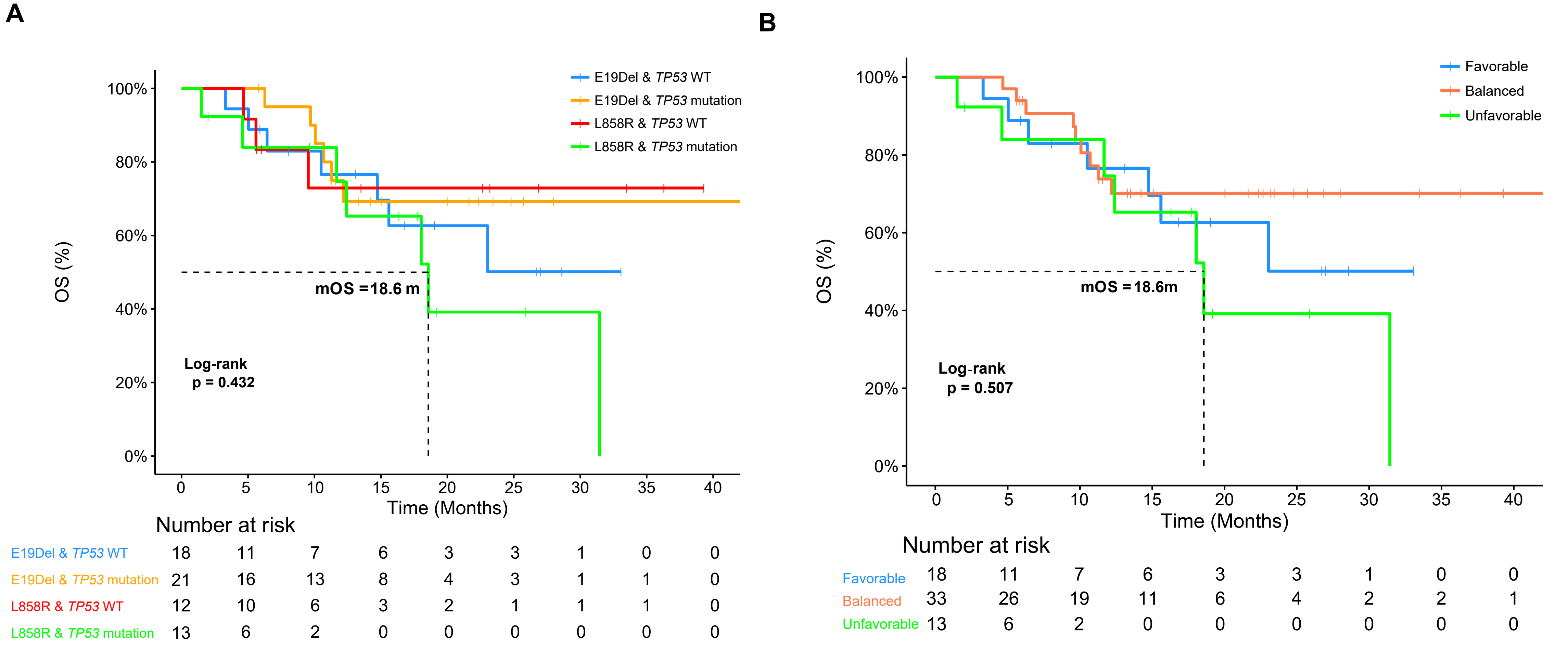
A
E19Del & TP53 WT
E19Del & TP53 mutation
L858R & TP53 WT
L858R & TP53 mutation
OS (%)
100%
80%
60%
40%
20%
0%
mOS =18.6 m
Log-rank
p = 0.432
0 5 10 15 20 25 30 35 40
Time (Months)
Number at risk
E19Del & TP53 WT 18 11 7 6 3 3 1 0 0
E19Del & TP53 mutation 21 16 13 8 4 3 1 1 0
L858R & TP53 WT 12 10 6 3 2 1 1 1 0
L858R & TP53 mutation 13 6 2 0 0 0 0 0 0
B
Favorable
Balanced
Unfavorable
OS (%)
100%
80%
60%
40%
20%
0%
mOS =18.6m
Log-rank
p = 0.507
0 5 10 15 20 25 30 35 40
Time (Months)
Number at risk
Favorable 18 11 7 6 3 3 1 0 0
Balanced 33 26 19 11 6 4 2 2 1
Unfavorable 13 6 2 0 0 0 0 0 0
