# Supplementary material for: Plasma‐Based Genomic Features Influencing Outcomes of T790M‐Positive Non–Small Cell Lung Cancer Receiving Osimertinib
Source: Cancer Med. 2025 Nov 12;14(21):e71319. doi: 10.1002/cam4.71319 (PMC12605980; doi:10.1002/cam4.71319)
Supplement: Supplementary file 4 — Table S1. Univariate Cox analysis of progression‐free survival and overall survival based on pretreatment clinical characteristics and genomic alterations detected in at least three patients among all patients (n = 64). [file CAM4-14-e71319-s007.docx]

| Table S1. Univariate Cox analysis of progression-free survival and overall survival based on pre-treatment clinical characteristics and genomic alterations detected in at least three patients among all patients (n=64). | | | | |
| --- | --- | --- | --- | --- |
| Characterastics | Progression-free survival | | Overall survival | |
|  | HR (95% CI) | p value | HR (95% CI) | p value |
| Age (Years)  ≥ 65 vs. <65 | 0.62 (0.31-1.23) | 0.168 | 1.82 (0.80-4.17) | 0.150 |
| Sex  Male vs. Female | 2.07 (1.12-3.83) | 0.018 | 2.03 (0.87-4.71) | 0.093 |
| Smoking  Ever vs. Never | 2.13 (1.12-4.06) | 0.018 | 3.00 (1.27-7.11) | 0.009 |
| CNS metastasis  Yes vs. No | 0.97 (0.46-2.05) | 0.931 | 1.41 (0.55-3.59) | 0.475 |
| Liver metastasis  Yes vs. No | 1.67 (0.70-4.03) | 0.245 | 3.13 (1.21-8.12) | 0.013 |
| Bone metastasis  Yes vs. No | 0.99 (0.54-1.79) | 0.970 | 2.13 (0.89-3.97) | 0.081 |
| Lymph node metastasis  Yes vs. No | 1.77 (0.84-3.75) | 0.128 | 1.23 (0.42-3.65) | 0.707 |
| Pleural effusion  With vs. Without | 1.16 (0.64-2.12) | 0.626 | 0.77 (0.34-1.74) | 0.525 |
| *EGFR* mutation  L858R vs. E19Del | 1.66 (0.90-3.04) | 0.099 | 1.27 (0.56-2.91) | 0.570 |
| *EGFR* T790M in plasma  Positive vs. Negative | 1.76 (0.54-5.71) | 0.344 | 3.35 (0.45-24.9) | 0.209 |
| *TP53* Mutation vs. WT | 1.94 (1.04-3.63) | 0.034 | 1.16 (0.51-2.65) | 0.728 |
| *EGFR* other mutations  Mutation vs. WT | 0.83 (0.35-1.98) | 0.681 | 1.02 (0.30-3.44) | 0.980 |
| *ARID1A* Mutation vs. WT | 1.96 (0.76-5.01) | 0.155 | 1.14 (0.27-4.90) | 0.859 |
| *CTNNB1* Mutation vs. WT | 0.49 (0.15-1.59) | 0.226 | 1.23e-08 (0-Inf) | 0.095 |
| *APC* Mutation vs. WT | 1.81 (0.64-5.14) | 0.257 | 1.40 (0.33-5.98) | 0.652 |
| *CYP2D6* Mutation vs. WT | 1.42 (0.51-3.99) | 0.503 | 1.28 (0.30-5.47) | 0.742 |
| *PKHD1* Mutation vs. WT | 1.26 (0.39-4.09) | 0.700 | 2.10 (0.49-8.99) | 0.308 |
| *PIK3CA* Mutation vs. WT | 2.77 (0.82-9.34) | 0.087 | 0.92 (0.12-6.93) | 0.938 |
| *BRCA2* Mutation vs. WT | 1.23 (0.38-3.99) | 0.732 | 0.79 (0.11-5.87) | 0.815 |
| *MED12*  Mutation vs. WT | 1.58 (0.56-4.45) | 0.382 | 0.71 (0.09-5.28) | 0.734 |
| *GRIN2A*  Mutation vs. WT | 1.03 (0.32-3.35) | 0.958 | 0.78 (0.10-5.80) | 0.805 |
| *RB1* Mutation vs. WT | 2.73 (0.82-9.05) | 0.088 | 1.46 (0.19-11.00) | 0.711 |
| *GNAS*  Mutation vs. WT | 0.72 (0.17-3.03) | 0.648 | 1.58 (0.37-6.80) | 0.534 |
| *BCR*  Mutation vs. WT | 0.88 (0.21-3.74) | 0.857 | 2.14 (0.49-9.31) | 0.297 |
| *EGFR* CNV  Amplification vs. WT | 0.97 (0.38-2.48) | 0.955 | 1.55 (0.46-5.25) | 0.478 |
| *NKX2-1* CNV  Amplification vs. WT | 1.11 (0.39-3.10) | 0.849 | 0.98 (0.23-4.20) | 0.977 |
| *MYC* CNV  Amplification vs. WT | 2.9 (0.88-9.55) | 0.065 | 4.37 (1.00-19.1) | 0.032 |

Abbreviations: WT, wild-type; HR, hazard ratio; CI, confidence interval; CNS, central nervous system; CNV, copy number variation; vs., versus.
