# Supplementary material for: Plasma‐Based Genomic Features Influencing Outcomes of T790M‐Positive Non–Small Cell Lung Cancer Receiving Osimertinib
Source: Cancer Med. 2025 Nov 12;14(21):e71319. doi: 10.1002/cam4.71319 (PMC12605980; doi:10.1002/cam4.71319)
Supplement: Supplementary file 5 — Table S2. Univariate Cox analysis of progression‐free survival and overall survival in plasma T790M‐positive patients according to baseline clinical characteristics and genomic alterations (n = 57). [file CAM4-14-e71319-s004.docx]

| Table S2. Univariate Cox analysis of progression-free survival and overall survival in plasma T790M-positive patients according to baseline clinical characteristics and genomic alterations (n=57). | | | | |
| --- | --- | --- | --- | --- |
| Characterastics | Progression-free survival | | Overall survival | |
|  | HR (95% CI) | p value | HR (95% CI) | p value |
| Age (Years)  ≥ 65 vs. <65 | 0.61 (0.30-1.24) | 0.167 | 2.02 (0.87-4.70) | 0.094 |
| Sex  Male vs. Female | 1.84 (0.97-3.48) | 0.059 | 2.07 (0.87-4.91) | 0.091 |
| Smoking  Ever vs. Never | 2.05 (1.06-3.94) | 0.029 | 2.75 (1.14-6.62) | 0.019 |
| CNS metastasis  Yes vs. No | 0.90 (0.42-1.92) | 0.786 | 1.24 (0.48-3.2) | 0.657 |
| Liver metastasis  Yes vs. No | 1.59 (0.66-3.87) | 0.298 | 2.86 (1.09-7.49) | 0.025 |
| Bone metastasis  Yes vs. No | 0.87 (0.47-1.63) | 0.667 | 1.84 (0.74-4.55) | 0.183 |
| Lymph node metastasis  Yes vs. No | 1.68 (0.79-3.58) | 0.175 | 1.10 (0.37-3.29) | 0.860 |
| Pleural effusion  With vs. Without | 1.29 (0.69-2.39) | 0.421 | 0.93 (0.40-2.15) | 0.864 |
| *EGFR* mutation  L858R vs. E19Del | 1.76 (0.94-3.31) | 0.073 | 1.33 (0.57-3.08) | 0.507 |
| Concomitant mutations  Yes vs. No | 0.78 (0.33-1.88) | 0.583 | 1.84 (0.74-4.55) | 0.183 |
| *TP53* Mutation vs. WT | 1.88 (0.95-3.7) | 0.065 | 0.93 (0.40-2.19) | 0.869 |
| *EGFR* other mutations  Mutation vs. WT | 0.78 (0.33-1.86) | 0.575 | 0.91 (0.27-3.09) | 0.878 |
| *ARID1A* Mutation vs. WT | 1.85 (0.72-4.75) | 0.196 | 1.04 (0.24-4.48) | 0.957 |
| *CTNNB1* Mutation vs. WT | 0.60 (0.18-1.96) | 0.393 | 1.21e-08 (0-Inf) | 0.114 |
| *APC* Mutation vs. WT | 1.72 (0.60-4.9) | 0.305 | 1.27 (0.30-5.47) | 0.746 |
| *CYP2D6* Mutation vs. WT | 1.34 (0.48-3.79) | 0.575 | 1.16 (0.27-4.97) | 0.846 |
| *PKHD1* Mutation vs. WT | 1.19 (0.36-3.86) | 0.776 | 1.89 (0.44-8.14) | 0.384 |
| *PIK3CA* Mutation vs. WT | 2.55 (0.75-8.65) | 0.119 | 0.862(0.11-6.50) | 0.885 |
| *BRCA2* Mutation vs. WT | 1.15 (0.35-3.77) | 0.812 | 0.71 (0.09-5.30) | 0.736 |
| *MED12*  Mutation vs. WT | 1.48 (0.52-4.18) | 0.459 | 0.65 (0.09-4.83) | 0.667 |
| *GRIN2A*  Mutation vs. WT | 0.96 (0.30-3.14) | 0.951 | 0.71 (0.10-5.35) | 0.742 |
| *RB1* Mutation vs. WT | 2.54 (0.76-8.47) | 0.117 | 1.34 (0.18-10.20) | 0.775 |
| *GNAS*  Mutation vs. WT | 0.69 (0.16-2.90) | 0.606 | 1.43 (0.33-6.16) | 0.632 |
| *BCR*  Mutation vs. WT | 0.85 (0.20-3.61) | 0.820 | 1.93 (0.44-8.44) | 0.372 |
| *EGFR* CNV  Amplification vs. WT | 0.93 (0.36-2.37) | 0.874 | 1.40 (0.41-4.78) | 0.585 |
| *NKX2-1* CNV  Amplification vs. WT | 1.05 (0.37-2.97) | 0.921 | 0.88 (0.21-3.79) | 0.865 |
| *MYC* CNV  Amplification vs. WT | 2.7 (0.82-8.92) | 0.090 | 3.97 (0.90-17.40) | 0.049 |

Abbreviations: WT, wild-type; HR, hazard ratio; CI, confidence interval; CNS, central nervous system; Inf, infinity; CNV, copy number variation; vs., versus.
