# Supplementary material for: Plasma‐Based Genomic Features Influencing Outcomes of T790M‐Positive Non–Small Cell Lung Cancer Receiving Osimertinib
Source: Cancer Med. 2025 Nov 12;14(21):e71319. doi: 10.1002/cam4.71319 (PMC12605980; doi:10.1002/cam4.71319)
Supplement: Supplementary file 6 — Table S3. Univariate Cox analysis of progression‐free survival and overall survival by establishing consecutive bTMB cut‐off points. [file CAM4-14-e71319-s005.docx]

| \| Table S3. Univariate Cox analysis of progression-free survival and overall survival by establishing consecutive bTMB cut-off points. \| \| \| \| \| \| --- \| --- \| --- \| --- \| --- \| \| Cut-points (mut./Mb) \| Progression-free survival \| \| Overall survival \| \| \| HR (95% CI) \| p value \| HR (95% CI) \| p value \| \| bTMB ≥10 vs. <10 \| 1.76 (0.78-4.02) \| 0.170 \| 2.35 (0.86-6.44) \| 0.087 \| \| bTMB ≥9 vs. <9 \| 1.60 (0.73-3.50) \| 0.236 \| 1.93 (0.70-5.29) \| 0.195 \| \| bTMB ≥8 vs. <8 \| 2.18 (1.08-4.44) \| 0.027 \| 2.05 (0.79-5.33) \| 0.134 \| \| bTMB ≥7 vs. <7 \| 1.62 (0.84-3.13) \| 0.150 \| 1.69 (0.69-4.14) \| 0.248 \| \| bTMB ≥6 vs. <6 \| 1.15 (0.63-2.11) \| 0.653 \| 1.05 (0.44-2.49) \| 0.920 \| \| bTMB ≥5 vs. <5 \| 1.29 (0.72-2.34) \| 0.394 \| 1.04 (0.46-2.39) \| 0.924 \| \| bTMB ≥4 vs. <4 \| 1.42 (0.78-2.56) \| 0.249 \| 1.23 (0.54-2.80) \| 0.622 \| \| bTMB ≥3 vs. <3 \| 1.58 (0.86-2.91) \| 0.136 \| 1.24 (0.54-2.84) \| 0.613 \| \| bTMB ≥2 vs. <2 \| 1.50 (0.77-2.92) \| 0.235 \| 1.32 (0.52-3.36) \| 0.554 \| \| bTMB ≥1 vs. <1 \| 1.67 (0.74-3.74) \| 0.212 \| 1.7 5(0.52-5.91) \| 0.359 \| |
| --- | --- | --- | --- | --- | --- | --- | --- | --- | --- | --- | --- | --- | --- | --- | --- | --- | --- | --- | --- | --- | --- | --- | --- | --- | --- | --- | --- | --- | --- | --- | --- | --- | --- | --- | --- | --- | --- | --- | --- | --- | --- | --- | --- | --- | --- | --- | --- | --- | --- | --- | --- | --- | --- | --- | --- | --- | --- | --- | --- | --- | --- | --- | --- | --- |

Abbreviations: HR, hazard ratio; CI, confidence interval; bTMB, blood tumor mutational burden; mut./Mb, mutations per megabase; vs., versus.
