# Supplementary material for: Plasma‐Based Genomic Features Influencing Outcomes of T790M‐Positive Non–Small Cell Lung Cancer Receiving Osimertinib
Source: Cancer Med. 2025 Nov 12;14(21):e71319. doi: 10.1002/cam4.71319 (PMC12605980; doi:10.1002/cam4.71319)
Supplement: Supplementary file 7 — Table S4. Univariate Cox analysis of progression‐free survival and overall survival by establishing consecutive bTMB cut‐off points in plasma T790M‐positive patients (n = 57). [file CAM4-14-e71319-s001.docx]

| \| Table S4. Univariate Cox analysis of progression-free survival and overall survival by establishing consecutive bTMB cut-off points in plasma T790M-positive patients (n=57). \| \| \| \| \| \| --- \| --- \| --- \| --- \| --- \| \| Cut-points (mut./Mb) \| Progression-free survival \| \| Overall survival \| \| \| HR (95% CI) \| p value \| HR (95% CI) \| p value \| \| bTMB ≥10 vs. <10 \| 1.66 (0.72-3.81) \| 0.226 \| 2.13 (0.77-5.89) \| 0.135 \| \| bTMB ≥9 vs. <9 \| 1.49 (0.68-3.30) \| 0.317 \| 1.74 (0.63-4.81) \| 0.281 \| \| bTMB ≥8 vs. <8 \| 2.05 (1.00-4.22) \| 0.046 \| 1.86 (0.71-4.88) \| 0.202 \| \| bTMB ≥7 vs. <7 \| 1.51 (0.77-2.97) \| 0.224 \| 1.51 (0.61-3.74) \| 0.370 \| \| bTMB ≥6 vs. <6 \| 1.05 (0.56-1.98) \| 0.874 \| 1.04 (0.43-2.50) \| 0.927 \| \| bTMB ≥5 vs. <5 \| 1.18 (0.64-2.18) \| 0.602 \| 1.00 (0.43-2.33) \| 0.996 \| \| bTMB ≥4 vs. <4 \| 1.30 (0.70-2.40) \| 0.406 \| 1.19 (0.51-2.76) \| 0.686 \| \| bTMB ≥3 vs. <3 \| 1.45 (0.77-2.75) \| 0.251 \| 1.16 (0.49-2.72) \| 0.740 \| \| bTMB ≥2 vs. <2 \| 1.36 (0.66-2.80) \| 0.408 \| 0.92 (0.36-2.35) \| 0.856 \| \| bTMB ≥1 vs. <1 \| 1.78 (0.69-4.55) \| 0.224 \| 1.11 (0.33-3.75) \| 0.869 \| |
| --- | --- | --- | --- | --- | --- | --- | --- | --- | --- | --- | --- | --- | --- | --- | --- | --- | --- | --- | --- | --- | --- | --- | --- | --- | --- | --- | --- | --- | --- | --- | --- | --- | --- | --- | --- | --- | --- | --- | --- | --- | --- | --- | --- | --- | --- | --- | --- | --- | --- | --- | --- | --- | --- | --- | --- | --- | --- | --- | --- | --- | --- | --- | --- | --- |

Abbreviations: HR, hazard ratio; CI, confidence interval; bTMB, blood tumor mutational burden; mut./Mb, mutations per megabase; vs., versus.
