# Supplementary material for: Plasma‐Based Genomic Features Influencing Outcomes of T790M‐Positive Non–Small Cell Lung Cancer Receiving Osimertinib
Source: Cancer Med. 2025 Nov 12;14(21):e71319. doi: 10.1002/cam4.71319 (PMC12605980; doi:10.1002/cam4.71319)
Supplement: Supplementary file 8 — Table S5. Univariate and multivariable Cox regression analysis of progression‐free survival and overall survival by pretreatment clinical and genomic characteristics in plasma T790M‐positive patients (n = 57). [file CAM4-14-e71319-s008.docx]

Table S5. Univariate and multivariable Cox regression analysis of progression-free survival and overall survival by pre-treatment clinical and genomic characteristics in plasma T790M-positive patients (n=57).

| Characteristics | Progression-free survival | | | | | | Overall survival | | | | |
| --- | --- | --- | --- | --- | --- | --- | --- | --- | --- | --- | --- |
|  | Univariate analysis | | | Multivariable analysis | | | Univariate analysis | | | Multivariable analysis | |
|  | HR (95% CI) | p value | HR (95% CI) | | p value | HR (95% CI) | | p value | HR (95% CI) | | p value |
| Age (Years)  ≥ 65 vs. <65 | - | - | - | | - | 2.02 (0.87-4.70) | | 0.094 | 2.96 (1.10-7.98) | | **0.032** |
| Sex | 1.84 (0.97-3.48) | 0.059 | 1.33 (0.32-5.62) | | 0.694 | 2.07 (0.87-4.91) | | 0.091 | 0.80 (0.10-6.50) | | 0.835 |
| Male vs. Female |  |  |  |  |  |  |  |  |  |  |  |
| Smoking history | 2.05 (1.06-3.94) | 0.029 | 1.59 (0.36-7.00) | | 0.540 | 2.75 (1.14-6.62) | | 0.019 | 4.20 (0.46-38.28) | | 0.203 |
| Ever vs. Never |  |  |  |  |  |  |  |  |  |  |  |
| *EGFR* driver | 1.76 (0.94-3.31) | 0.073 | 2.86 (1.29-6.34) | | **0.009** | - | | - | - | | - |
| L858R vs. E19Del |  |  |  |  |  |  |  |  |  |  |  |
| Bone metastasis | - | - | - | | - | 2.13 (0.89-3.97) | | 0.081 | 1.24 (0.46-3.39) | | 0.669 |
| Yes vs. No |  |  |  |  |  |  |  |  |  |  |  |
| Liver metastasis | - | - | - | | - | 2.86 (1.09-7.49) | | 0.025 | 2.43 (0.84-7.03) | | 0.101 |
| Yes vs. No |  |  |  |  |  |  |  |  |  |  |  |
| *TP53* | 1.88 (0.95-3.7) | 0.065 | 2.32 (1.07-5.03) | | **0.034** | - | | - | - | | - |
| Mutation vs. WT |  |  |  |  |  |  |  |  |  |  |  |
| *PIK3CA* | - | - | - | | - | - | | - | - | | - |
| Mutation vs. WT |  |  |  |  |  |  |  |  |  |  |  |
| *RB1* | - | - | - | | - | - | | - | - | | - |
| Mutation vs. WT |  |  |  |  |  |  |  |  |  |  |  |
| *CTNNB1* | - | - | - | | - | - | | - | - | | - |
| Mutation vs. WT |  |  |  |  |  |  |  |  |  |  |  |
| *MYC* CNV | 2.7 (0.82-8.92) | 0.090 | 1.19 (0.25-5.6) | | 0.824 | 3.97 (0.90-17.40) | | 0.049 | 3.01 (0.54-16.86) | | 0.210 |
| Amplification vs. WT |  |  |  |  |  |  |  |  |  |  |  |
| bTMB (mut./Mb) | 2.05 (1.00-4.22) | 0.046 | 1.62 (0.64-4.11) | | 0.310 | - | | - | - | | - |
| ≥8 vs. <8 |  |  |  |  |  |  |  |  |  |  |  |

Abbreviations: HR, hazard ratio; CI, confidence interval; E19Del, exon 19 deletion; WT, wild-type; CNV, copy number variation; Inf, infinity; bTMB, blood tumor mutational burden; mut./Mb, mutations per megabase; vs., versus.
